# Development of SNP markers for genes of the phenylpropanoid pathway and their association to kernel and malting traits in barley

**DOI:** 10.1186/1471-2156-14-97

**Published:** 2013-10-02

**Authors:** Manuela Peukert, Stephan Weise, Marion S Röder, Inge E Matthies

**Affiliations:** 1 Leibniz-Institute of Plant Genetics and Crop Plant Research (IPK), OT Gatersleben, Corrensstr. 3, 06466 Stadt Seeland, Germany

**Keywords:** Phenylpropanoids, Barley, SNP, Haplotype, Associations, Malting quality parameters

## Abstract

**Background:**

Flavonoids are an important class of secondary compounds in angiosperms. Next to certain biological functions in plants, they play a role in the brewing process and have an effect on taste, color and aroma of beer. The aim of this study was to reveal the haplotype diversity of candidate genes involved in the phenylpropanoid biosynthesis pathway in cultivated barley varieties (*Hordeum vulgare* L.) and to determine associations to kernel and malting quality parameters.

**Results:**

Five genes encoding phenylalanine ammonia-lyase (*PAL*), cinnamate 4-hydroxylase (*C4H*), chalcone synthase (*CHS*), flavanone 3-hydroxylase (*F3H*) and dihydroflavonol reductase (*DFR*) of the phenylpropanoid biosynthesis pathway were partially resequenced in 16 diverse barley reference genotypes. Their localization in the barley genome, their genetic structure, and their genetic variation e.g. single nucleotide polymorphism (SNP) and Insertion/Deletion (InDel) patterns were revealed. In total, 130 SNPs and seven InDels were detected. Of these, 21 polymorphisms were converted into high-throughput pyrosequencing markers. The resulting SNP and haplotype patterns were used to calculate associations with kernel and malting quality parameters.

**Conclusions:**

SNP patterns were found to be highly variable for the investigated genes. The developed high-throughput markers are applicable for assessing the genetic variability and for the determination of haplotype patterns in a set of barley accessions. The candidate genes *PAL*, *C4H* and *F3H* were shown to be associated to several malting properties like glassiness (*PAL*), viscosity (*C4H*) or to final attenuation (*F3H*).

## Background

In 2010, Germany ranked 1^st^ with 10,412,100 tons of global barley production followed by France, Ukraine and Canada [1]. For human consumption barley is mostly supplied to brewing and distilling [2], but recently, the interest in barley as a functional food increased due to its content of beneficial components for the human diet [3-6]. Thus, important breeding aims are to enhance the malting quality next to the improvement of yield components.

**Figure 1:**
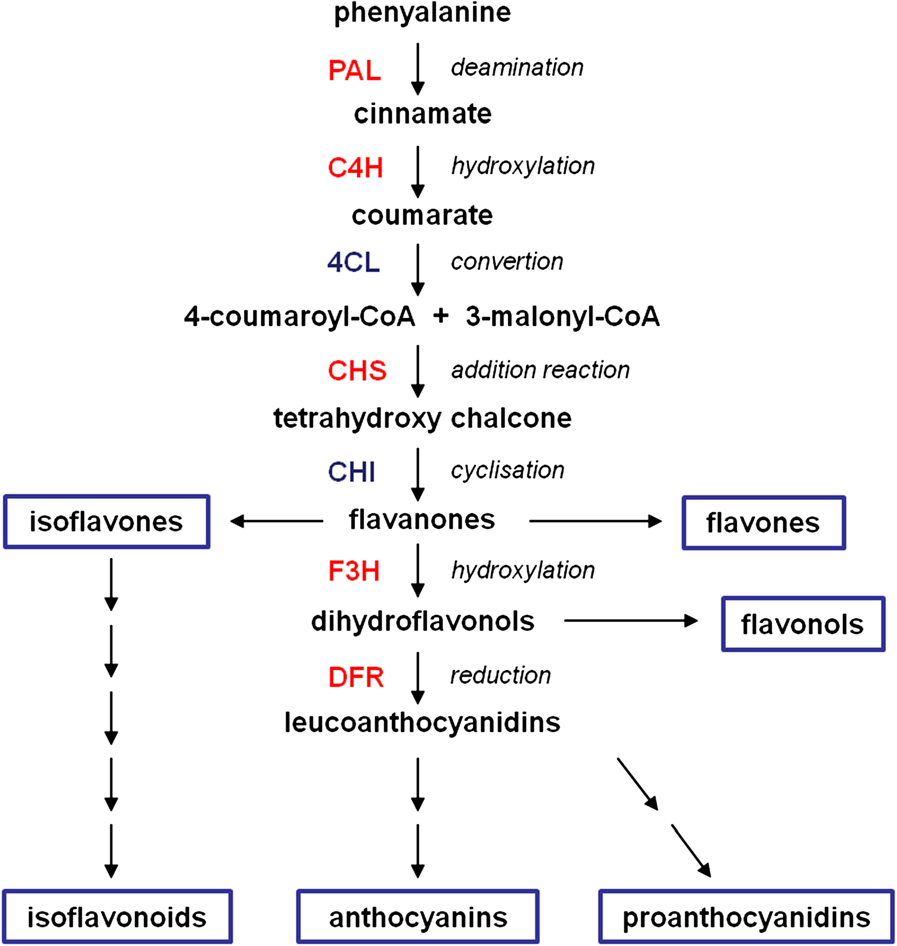
**Biosynthetic pathway of flavonoids with the investigated genes labeled in red.** Abbreviations of the enzymes are: PAL – phenylalanine ammonia-lyase; C4H – cinnamate 4-hydroxylase; 4CL – 4-coumaroyl-CoA ligase; CHS – chalcone synthase; CHI – chalcone isomerase; F3H – flavanone 3-hydroxylase; DFR – dihydroflavonol reductase.

All genes studied here are part of the phenylpropanoid pathway, coding especially for enzymes of the flavonoid synthesis pathway. Their gene products represent the mostly abundant group of secondary metabolites in angiosperms [7]. A wide range of phenylpropanoids is abundant in the barley grain, such as phenolic acids and flavanols (such as proanthocyanidins) [3]. The heterogeneous group of flavonoids is derived from phenylalanine and malonyl-CoA. Several classes of them can be differentiated by the oxidation states of the carbon atoms C-2, C-3 and C-4 in the oxygenic heterocycle, and they are mainly stored in plant vacuoles. The corresponding enzymes of biosynthesis are assumed to form membrane-associated complexes at the cytoplasmatic oriented side of the rough endoplasmatic reticulum [8]. A general overview of the biosynthetic pathway is given in Figure 1. Starting from the general phenylpropanoid metabolism phenylalanine is deaminated to cinnamate catalyzed by the phenylalanine ammonia-lyase (PAL). The cinnamate 4-hydroxylase (C4H) hydroxylates the product to coumarate, which is then converted to 4-coumaroyl-CoA. As a first step in the flavonoid pathway one molecule 4-coumaroyl-CoA is added to three molecules malonyl-CoA to form tetrahydroxy chalcone, catalyzed by the chalcone synthase (CHS). The following steps to the formation of anthocyanins include the chalcone isomerase (CHI) catalyzed cyclization of naringenin chalcone to naringenin (flavanone), the production of dihydroflavonols by the flavanone 3-hydroxylase (F3H), and the reduction of them to leucoanthocyanidins by the dihydroflavonol reductase (DFR) [9]. Other branches of the central biosynthesis lead to the formation of flavones, isoflavonoids or flavonols.

The high structural diversity of flavonoids is related to many biological functions: Anthocyanins are plant pigments and serve as attractants for pollinators and seed dispersers. Others flavonoids are known to exhibit repellent functions against herbivorous insects [10]. In form of phytoalexines they possess antimicrobial effects. Abiotic stress factors such as salt, low temperatures or high light intensities enhance the flavonoid content in plant or grain tissues [11], which is related to the antioxidant capacities of these compounds. The property of phenols to act as scavengers of free radicals such as reactive oxygen species [6] constitutes to the importance of flavonoids as pharmacological substances effecting cancer, cardiovascular and age-related degenerative diseases [5,12]. The antioxidant effects play a role during the malting and brewing process as well, where barley flavonoids have an impact on taste, color and foam stability of the beer [5,6]. A high content of proanthocyanidins causes precipitation of proteins in beer resulting in formation of colloidal haze [13]. Proanthocyanidine free barley accessions possess same malting properties and show better chemical and physical stability of the beer, but a negative influence on the flavor stability was observed [14]. Additionally, the use of barley accessions with a high content of condensed proanthocyanidins (tannins) lead to a more intensive coloration during the brewing process.

The relationship between genetic diversity and phenotypic performance is assessed by association studies. Source for these investigations are single nucleotide polymorphisms (SNP) and the combination of various SNPs within one gene to haplotypes. Several association studies for specific candidate genes were performed in barley [15-21]. Until now, no genes from the secondary phenylpropanoid pathway were investigated for their impact on malting traits.

The aim of the present study was (i) the assessment of allelic diversity of genes representing the phenylpropanoid pathway in barley and (ii) the determination of significant associations of the detected single nucleotide polymorphisms (SNPs) or their resulting haplotypes with kernel and malting quality parameters.

## Results and discussion

### SNP Patterns and marker development

Five genes of the phenylpropanoid metabolic pathway were investigated for their abundance of polymorphisms and for associations to kernel and malting quality parameters, which will be further described.

A high variability of SNP frequency in the investigated fragments of the candidate genes was observed (Table 1). The highest number of detected SNPs was observed in one fragment of the *PAL* encoding gene. For PAL_2 a frequency of 58.8 SNPs/kb was found. Less SNPs were detected in the gene fragment CHS_GM290 with a density of 1.5 SNPs/kb. This high polymorphic variability was also described in the findings of Bundock et al., Kanazin et al. and Rostoks et al. [22-24]. Additionally, Matthies et al. [19-21] revealed highly different SNP-frequencies in candidate genes of the C-metabolism in barley.

**Table 1:** Genetic structure of all investigated candidate gene fragments of the phenylpropanoid pathway after resequencing and aligning of 16 barley reference genotypes

**Gene fragment**	**Fragment size [bp]**	**Exon size [bp]**	**Intron size [bp]**	**3' UTR [bp]**	**No. of InDels**	**No. of SNPs**	**SNP frequency per kb**
PAL_1	604	604	-	-	0	5	8.3
PAL_2	595	595	-	-	0	35	58.8
C4H_1	495	46	-	449	0	4	8.1
C4H_4	681	681	-	-	0	2	2.9
CHS_1	252	252	-	-	0	0	0
CHS_2	255	255	-	-	0	0	0
CHS_3	369	369	-	-	0	0	0
CHS_GM0287	321	235	-	86	0	11	34.3
CHS_GM0290	661	661	-	-	0	1	1.5
CHS_GM0293	474	474	-	-	0	17	35.9
F3H_1	796	199	597	-	4	48	60.4
F3H_GM022	544	389	109	46	2	3	5.5
DFR_1	618	428	105	85	0	1	1.6
DFR_3	564	459	105	-	1	3	5.3
DFR_4	915	759	105	51	1	3	3.3

#### Phenylalanine ammonia-lyase (PAL)

**Figure 2:**
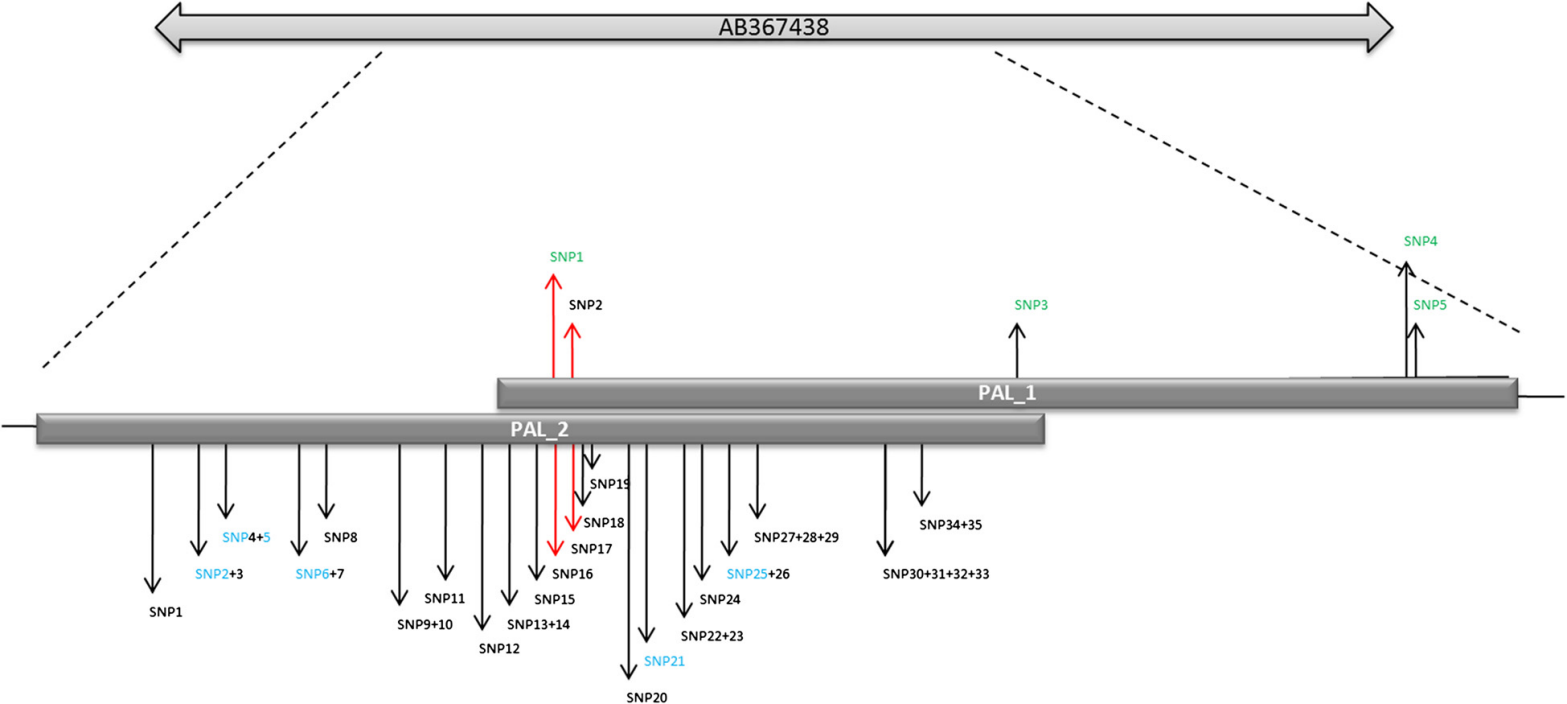
**Structures of the sequenced phenylalanine ammonia**-**lyase** (***PAL***) **gene fragments PAL**_**1 and PAL**_**2.** Comparison of both fragments revealed differing SNP patterns indicating to belong to different gene copies of the *PAL* gene family. Exons are depicted as light grey boxes. Blue – CAPS marker available, green – high-throughput SNP marker developed, red arrows – shared SNP-positions of both investigated gene fragments.

Two fragments of the gene encoding *PAL* were amplified resulting in a total of 877 bp of the sequenced exonic region. Both fragments have an overlapping identical region of 322 bp. Within this area a completely different SNP pattern was observed with only two identical SNPs between both sequenced fragments (SNP 1 and SNP 2 of PAL_1 with SNPs 16 and 17 of PAL_2, illustrated in Figure 2). Thus, both fragments must belong to different gene copies of the *PAL* gene family. Kervinen et al. [25] also observed at least five copies of a *PAL* gene family in barley. The consensus sequences for both fragments generated from 16 reference genotypes were 99% identical to the cDNA clone AB367438 [26].

Five SNPs were identified in the 604 bp long fragment PAL_1. All represent silent mutations and defined three haplotypes (Additional file 1). Except of PAL_1_SNP2 all SNPs were converted into pyrosequencing markers for the use in high-throughput genotyping (Table 2). For SNP2 no distinct alleles were observed, which can be explained by non-specific annealing of the pyrosequencing primer to different *PAL* gene family members. The second gene fragment PAL_2 consists of 595 nucleotides with 35 detected SNPs in a set of 16 reference genotypes. Two of these detected SNPs were missense mutations. SNP2 causes an amino acid exchange of methionin (ATG) into isoleucine (ATA) and SNP24 leads to an exchange of leucin (CTC) into phenylalanine (TTC). Five out of 35 detected SNPs were converted into CAPS markers (Figure 2). Despite of the high SNP frequency (58.8 SNP/kb) a low number of haplotypes is stated due to linkage (Additional file 2). Two haplotypes (PAL_2_H3 and H4) possessed completely different SNP patterns while five haplotypes differed only in one of six SNPs (Additional file 2).

**Table 2:** **SNP and haplotype pattern of the phenylalanine ammonia**-**lyase** (***PAL***) **encoding gene**, **assessed by pyrosequencing assays for the SNPs 1**, **3**, **4 and 5 in a set of 190 European barley cultivars and the 6**-**rowed mapping parents Steptoe and Morex**

**Haplotypes**	**SNPs from 5' to 3'**				**Haplotype frequency (No. of cultivars) according row number and growth habit**				**Haplotype frequency (No. of cultivars)**
	**SNP1**	**SNP3**	**SNP4**	**SNP5**	**2r-S**	**6r-S**	**2r-W**	**6r-W**	
PAL_H1	A (Pro)	C (Leu)	A (Arg)	G (Ala)	80	–	38	3	121
PAL_H2	C (Pro)	C (Leu)	A (Arg)	G (Ala)	13	1	3	25	42
PAL_H3	C (Pro)	T (Leu)	T (Arg)	A (Ala)	1	1	20	6	28
Unknown	Missing SNP information				–	–	–	1	1
Total					94	2	61	35	192

All *SNP* markers shown in 5' to 3' direction are localized in the exonic region. Coding amino acids are given in brackets. *S* = Spring, *W* = Winter, 2r = two-rowed, 6r = six-rowed.

#### Cinnamate 4-hydroxylase (C4H)

**Figure 3:**
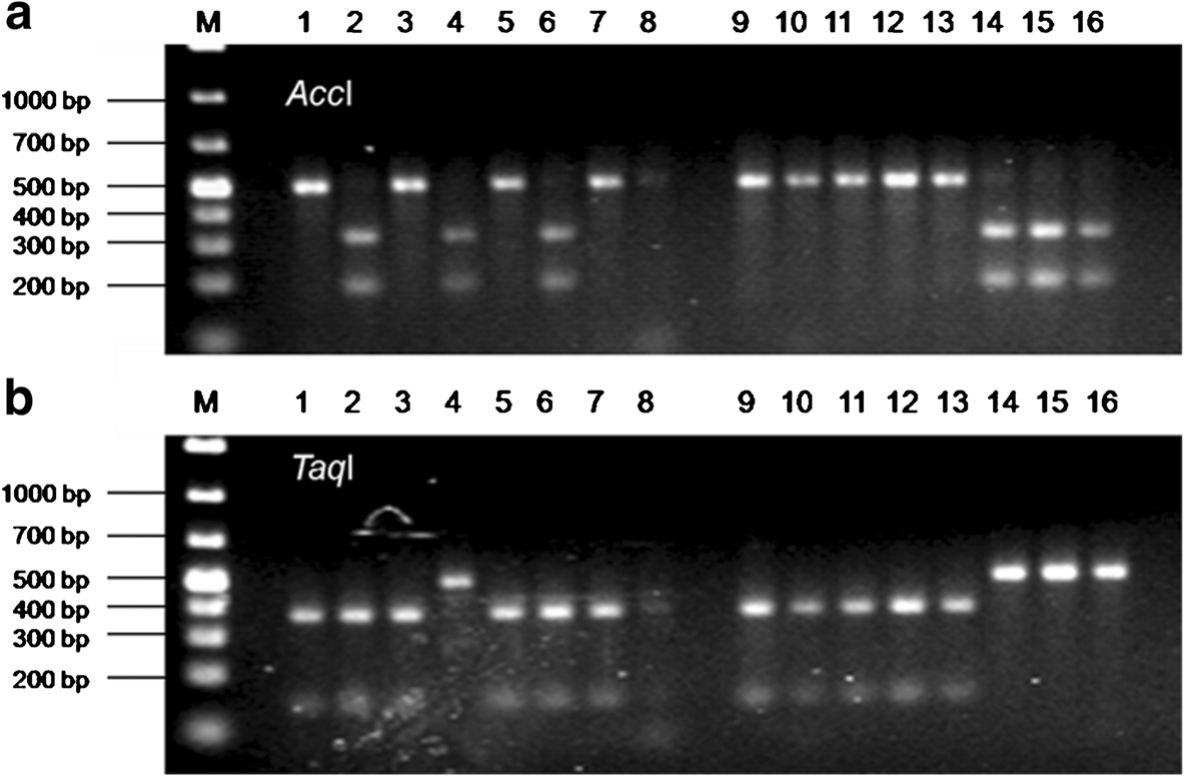
**CAPS assays for SNP 1 (a) and SNP 4 (b) of the C4H**_**1 gene fragment.** The restriction pattern is derived by using the enzymes *Acc*I for SNP1 and *Taq*I for SNP4, and is shown for 16 reference genotypes. M = 1 kb marker.

The two resequenced gene fragments from *C4H* showed 100% identity to cDNA AK250541 [27]. The first fragment C4H_4 with 681 bp is located near the 5’ end whereas C4H_1 consists of 46 bp exonic sequence followed by 449 bp 3’ UTR. Both fragments have no introns. Altogether, six SNPs were detected and four of them were converted into pyrosequencing markers (Additional file 3). CAPS assays for two of the SNP-markers are also available (Figure 3). The exonic polymorphisms C4H_4_SNP1 and C4H_4_SNP2 are silent mutations. Four SNPs found in C4H_1 are localized in the 3’UTR.

Genotyping for C4H_1 was performed with 190 barley cultivars using all four pyrosequencing markers. Three haplotypes were determined (Table 3).

**Table 3:** **SNP and haplotype pattern of cinnamate 4**-**hydroxylase** (***C4H***) **encoding gene**, **investigated with pyrosequencing** (**SNPs 1 to 4**) **depicted in 5**' **to 3**' **direction**

**Haplotypes**	**SNPs from 5' to 3'**				**Haplotype frequency (No. of cultivars) according row number and growth habit**				**Haplotype frequency (No. of cultivars)**
	**SNP1**	**SNP2**	**SNP3**	**SNP4**	**2r-S**	**6r-S**	**2r-W**	**6r-W**	
C4H_H1	T	C	A	A	86	1	38	1	126
C4H_H2	C	T	T	G	2	–	23	33	58
Unknown	Missing SNP information				6	1	–	1	8
Total					94	2	61	35	192

All four markers are localized in the 3'-non-coding region of the gene and were observed within a set of 190 European barley cultivars and the mapping parents Steptoe x Morex. *S* = Spring, *W* = Winter, 2r = two-rowed, 6r = six-rowed.

#### Chalcon synthase (CHS)

It is known that the *CHS* in *H*. *vulgare* represents a gene family [28]. Six fragments were amplified. The genomic sequences of CHS_1, CHS_2 and CHS_3 revealed no polymorphisms within the 16 reference genotypes. Two fragments CHS_GM290 and CHS_GM293 shared identical regions of the cDNA clone Y09233 [28], and comparable to the findings for the *PAL* gene fragments different SNP patterns were observed (Additional file 4). Therefore it is assumed that both fragments belong to different gene copies of the *CHS* gene family. For CHS_GM290 a 661 bp exonic sequence containing only one SNP was detected, which was converted into a pyrosequencing marker. This fragment showed 100% identity to the cDNA Y09233 [28]. The consensus sequence of CHS_GM293 derived from 16 reference genotypes showed 88% identity to Y09233 only. In this 474 bp fragment, 17 SNPs were detected and two of them were converted into pyrosequencing markers. Three of them caused amino acid substitutions (Additional file 5). SNP2 causes an exchange of lysine and arginine, while the adjacent SNP13 and SNP14 were coding for either leucine (CTC) or proline (CCT). Alleles of SNP16 and SNP17 are resulting in either asparagine (AAT) or aspartate (GAC). The fragment CHS_287 is located at the end of the gene and consists of 200 bp exon and 120 bp 3’UTR. After resequencing, 99% identity with cDNA U43494 [29] was revealed. Two of the 11 detected SNPs generate amino acid exchanges: SNP1 codes for arginine or cysteine and SNP2 for valine or alanine (Additional file 6). It is remarkable that all eight SNPs from GM287_SNP4 to GM287_SNP11 are very close to each other. The SNP1 was converted into a pyrosequencing marker and genotyping of 190 barley accessions was performed with this pyrosequencing marker CHS_GM287_SNP1 (Table 4).

**Table 4:** **Allelic frequency of SNP1 found in the chalcon synthase** (***CHS***) **encoding gene**, **in a set of 190 European barley cultivars and the mapping parents Steptoe and Morex**

**SNP1**	**Genotype frequency (No. of cultivars) according row number and growth habit**				**Haplotype frequency (No. of cultivars)**
**Exon**	**2r-S**	**6r-S**	**2r-W**	**6r-W**	
C (Arg)	94	1	53	24	172
T (Cys)	–	1	8	9	18
Missing SNP information	–	–	–	2	2
Total	94	2	61	35	192

Coding amino acids are given in brackets. *S* = Spring, *W* = Winter, 2r = two-rowed, 6r = six-rowed.

#### Flavone 3-hydroxylase (F3H)

**Figure 4:**
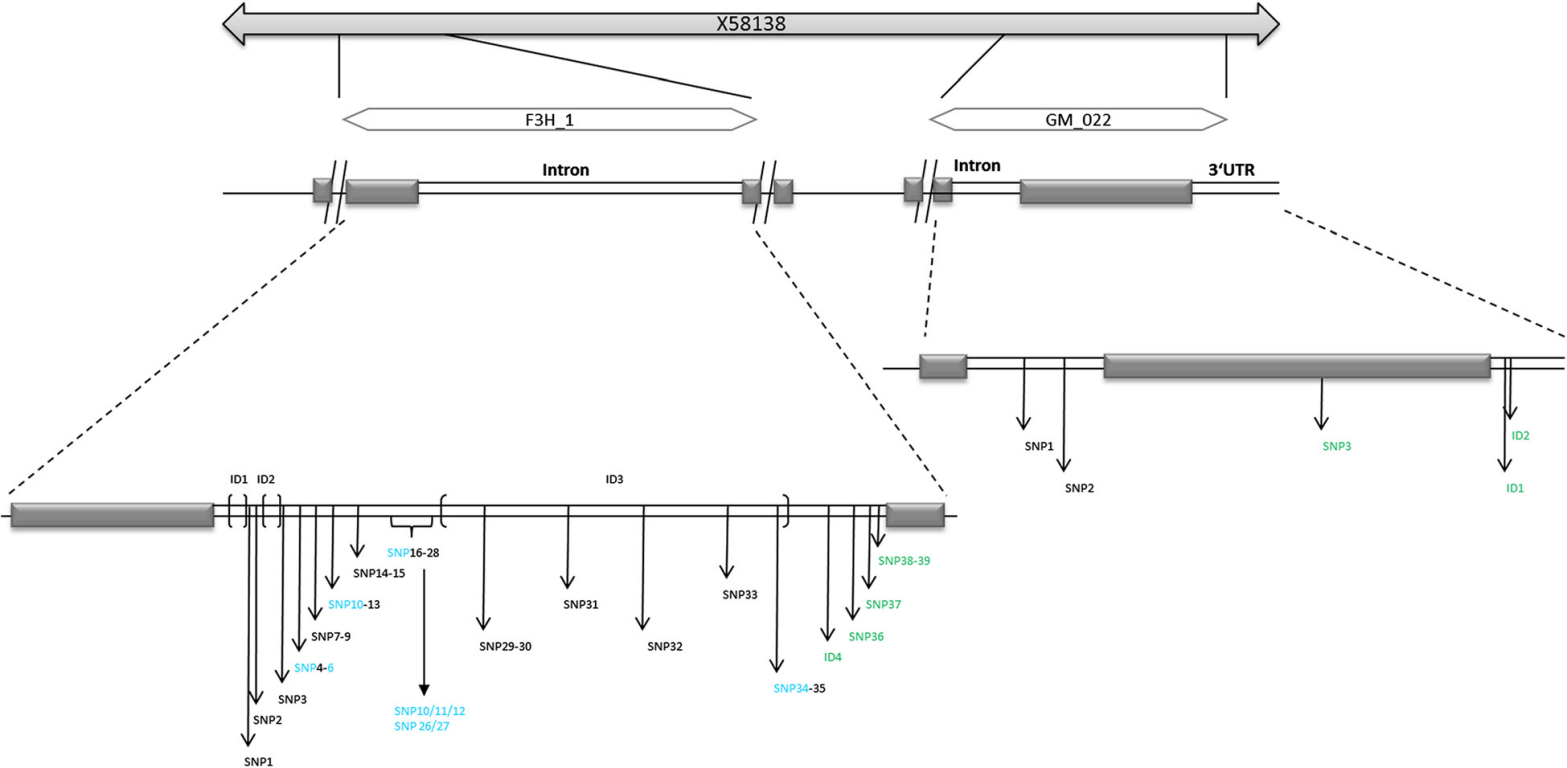
**Genetic structure of the flavanone 3-hydroxylase (*F3H*) gene fragments F3H_1 and GM022.** Light grey boxes represent exons and white boxes represent introns. The thin line indicates the non-sequenced region. ID – Insertion/Deletion, blue – CAPS marker, green – high-throughput SNP marker.

Two nearby fragments of the *F3H* with altogether 1339 bp were sequenced and analyzed. The first fragment F3H_1 is localized near the 5’-end of the gene and contains 167 bp of one exon and 597 bp of an intron. Here, 48 SNPs and four InDels were detected in the intron (Figure 4). Four of these SNPs (SNP36, 37, 38 and 39), as well as ID4, were converted into pyrosequencing markers while the large InDel ID3 of 296 bp length was detected by gel electrophoresis.

The second fragment F3H_GM022 with 544 bp length is localized at the 3’-end of the gene containing a 109 bp large intron flanked by a 37 bp exon sequence in 5’ direction and a 336 bp long exon sequence in 3’ direction comprising the stop-codon TAG. The 3’UTR region was partially sequenced as well (Figure 4). Three SNPs and two InDels were detected here. The first two SNPs are located in the intron while SNP3 is located in the 336 bp exon representing a silent mutation. Two InDels of three bp (ID1) and one bp (ID2) length were found in the 3’UTR. All polymorphisms of F3H_GM022 were converted into pyrosequencing markers.

All exon sequences of F3H_1 and GM022 showed high identity to the cDNA X58138 encoding the F3H amino acid sequence of barley [9]. The entire sequence of F3H_1 including the large intron showed no similarity to any published genetic sequences of *F3H* whereas the complete sequence of F3H_GM022 including the intron showed high similarity to *F3H* gene sequences e.g. AB223024 and AB223026 (http://www.ncbi.nlm.nih.gov) from *Triticum aestivum* (≥91%).

Genotyping was performed with 190 cultivars by applying high-througput marker assays for seven SNPs and two InDels. Both InDels of F3H_GM022 ID1 and ID2 were not included into the haplotype and association analysis due to unclear and rare allele pattern. At least, four haplotypes could be determined. The haplotype F3H_H1 is predominantly found in winter cultivars and the other three haplotypes F3H_H2, F3H_H3 and F3H_H4 are mainly abundant in the spring types (Table 5).

**Table 5:** **SNP and haplotype pattern in the adjacent fragments F3H**_**1 and GM022 of the flavanone 3**-**hydroxylase** (***F3H***) **encoding gene revealed by gel electrophoresis** (**296 bp InDel**) **and by pyrosequencing assays in a set of 190 European barley cultivars and the mapping parents Steptoe and Morex**

**Haplotypes**	**SNPs/IDs from 5' to 3'**									**Haplotype frequency (No. of cultivars) according row number and growth habit**				**Haplotype frequency (No. of cultivars)**
	**F3H1_ID3**	**F3H1_ID4**	**F3H1_SNP36**	**F3H1_SNP37**	**F3H1_SNP38**	**F3H1_SNP39**	**GM022_SNP1**	**GM022_SNP2**	**GM022_SNP3**	**2r-S**	**6r-S**	**2r-W**	**6r-W**	
	**Intron**	**Intron**	**Intron**	**Intron**	**Intron**	**Intron**	**Intron**	**Intron**	**Exon**					
F3H_H1	296 bp insert	–	C	C	C	G	C	C	C (Leu)	15	1	59	31	106
F3H_H2	–	AT	T	C	G	A	G	A	C (Leu)	29	1	1	1	32
F3H_H3	296 bp insert	–	C	C	G	G	G	A	T (Leu)	28	–	1	1	30
F3H_H4	–	AT	C	A	G	A	G	A	C (Leu)	13	–	–	–	13
Unknown	Missing SNP information									9		–	2	8
Total										94	2	61	35	192

Coding amino acids are given in brackets. *ID* = Insertion/Deletion, *S* = Spring, *W* = Winter, 2r = two-rowed, 6r = six-rowed.

#### Dihydoflavonol reductase (DFR)

The DFR gene was partially resequenced. The obtained fragment of 949 bp consists of 174 bp from the third exon, followed by a 105 bp large intron and 585 bp of the fourth and last exon with two stop codons, followed by the 3’UTR of 85 bp. The sizes of the intron and this exon are in accordance to the results from Kristiansen and Rohde [30]. Resequencing of this candidate gene was performed with three overlapping amplified fragments that were completely identical in their sequence. Altogether, four SNPs and one InDel were found (Additional file 7). The first two SNPs in the third exon display silent mutations. DFR_SNP2 to DFR_SNP4 and DFR_ID1 were converted into pyrosequencing markers. Only SNP4 and ID1 were used for the large scale genotyping due to a rare allele pattern of SNP_2. Four haplotypes resulting from these two polymorphisms were revealed (Table 6). Two haplotypes (DFR_H1 and DFR_H2) are predominantly represented by two-rowed varieties, whereas most of DFR_H3 is represented by six rowed varieties (Table 6). The haplotype DFR_H4 shows a rare allele pattern.

**Table 6:** **Haplotype pattern resulting from one InDel (ID1) and the SNP4 (in 5’ to 3’ direction) of the dihydoflavonol reductase ( *DFR *) encoding gene investigated by pyrosequencing assays in a set of 190 European barley cultivars and the mapping parents Steptoe and Morex**

**Haplotypes**	**SNPs from 5' to 3'**		**Haplotype frequency (No. of cultivars) according row number and growth habit**				**Haplotype frequency (No. of cultivars)**
	**ID1**	**SNP4**	**2r-S**	**6r-S**	**2r-W**	**6r-W**	
	**Intron**	**3'UTR**					
DFR_H1	AA	C	61	–	36	4	101
DFR_H2	–	C	21	–	16	–	37
DFR_H3	AA	T	7	–	6	24	37
DFR_H4	–	T	3	2	2	7	14
unknown	missing SNP information		2	–	1	–	3
Total			94	2	61	35	192

ID = Insertion/Deletion, *S* = Spring, *W* = Winter, 2r = two-rowed, 6r = six-rowed.

**Figure 5:**
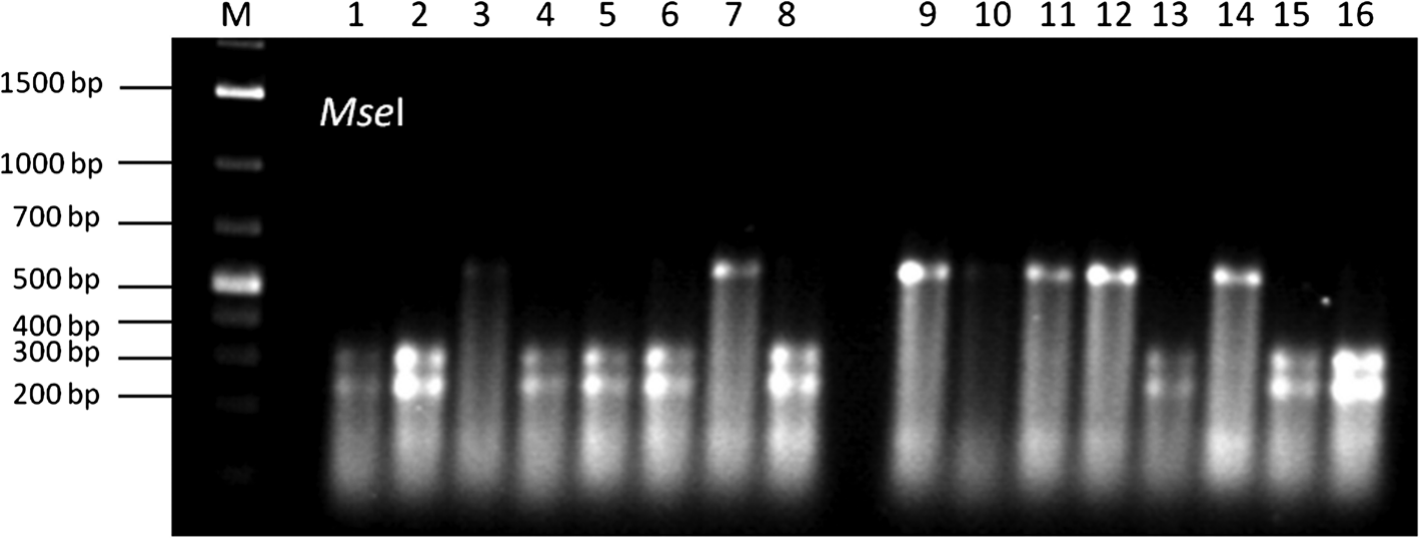
**Restriction pattern observed by the CAPS marker DFR_ ID1 developed for the two bp InDel in the dihydoflavonol reductase (*DFR*) encoding gene, shown for 16 reference genotypes. **M = 1 kb marker.

The InDel DFR_ID1 was transformed into a CAPS marker as well. A deletion of two adenine molecules at ID1 generates a recognition site for the restriction enzyme *Mse*I. Thus, all varieties with this deletion were cutted into fragments of 250 and 312 bp. This was also confirmed by the pyrosequencing assay for DFR_ID1 (Figure 5).

### Genetic mapping

Genetic mapping of *PAL*, *C4H*, *CHS* and *F3H* was performed using pyrosequencing markers found to be polymorphic between the mapping parents Steptoe x Morex on a set of 77 doubled haploid lines.

**Figure 6:**
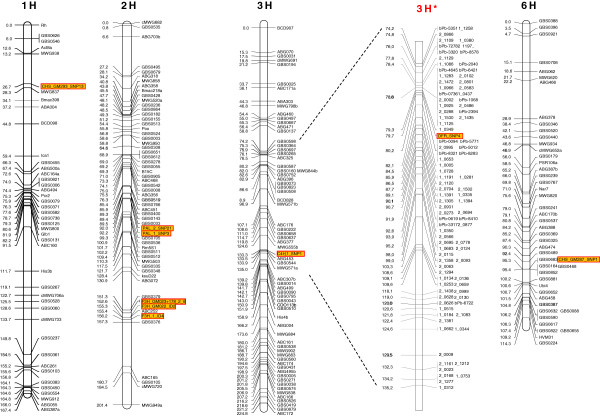
**Genetic mapping of the candidate genes from the Phenylpropanoid pathway: phenylalanine ammonia-lyase (*PAL*), cinnamate 4-hydroxylase (*C4H*), chalcone synthase (*CHS*), flavanone 3-hydroxylase (*F3H*) and dihydroflavonol reductase (*DFR*).** All gene fragments were mapped using the Steptoe x Morex mapping population, except *DFR* which was mapped on chromosome 3H* using the Morex x Barke population (kindly provided by Nils Stein, IPK Gatersleben).

For the *DFR* gene no polymorphism between Steptoe and Morex was found. Therefore, mapping was performed with the segregating population of Morex x Barke by Nils Stein (Leibniz Institute of Plant Genetics and Crop Plant Research, Gatersleben, Germany), calculated with a publicly available set of DArT and SNP markers [31,32]. This gene was mapped with the pyrosequencing marker DFR_SNP4, which is localized in the 3’UTR of the gene. It was localized between the DArT-marker bPb-0094 and the SNP-marker 1_0349 on the long arm of chromosome 3H, which are close to the centromeric region (Figure 6). Two markers for PAL_2, SNP21 detected by a CAPS-assay, and SNP3 by pyrosequencing, were mapped next to each other on chromosome 2H (Figure 6). Also for C4H_SNP1, mapping was performed using two methods (pyrosequencing and CAPS). Both could be placed at the same position on the long arm of 3H next to the RFLP marker ABG453 (1.3 cM) (Figure 6). SNPs of the three *CHS* fragments showed polymorphisms between the mapping parents Steptoe and Morex and were localized on different positions in the barley genome, respectively (Figure 6). This leads to the assumption that the three fragments belong to different gene copies. In previous studies from Christensen et al. [28] at least seven copies of *CHS* in the barley genome were identified but clear positioning of all these paralogs is still unclear. CHS_GM287_SNP1 was placed on the short arm of chromosome 6H linked to the marker GBS068. CHS_GM290_SNP1 was localized on the long arm of chromosome 1H, next to the marker Cab2, and CHS_GM293_SNP13 was mapped to the short arm of chromosome 1H, adjacent to the marker MWG837 (1.6 cM). The InDels of both fragments from *F3H* were mapped using the pyrosequencing markers F3H_1_ID4 and GM022_ID2. These were localized on the long arm of chromosome 2H, linked with the RFLP marker ABC252 (Figure 6), which is in accordance with the findings of Khlestkina et al. [33].

Here in this study, the genetic markers for *PAL*, *C4H*, *CHS* and *DFR* were mapped in the barley genome for the first time. Previous findings that *CHS* and *PAL* are represented by gene families were supported by mapping of non-overlapping markers to different positions in the barley genome (Figure 6). Location of *F3H* supports the results from Khlestkina et al. [33]. Recent advances in obtaining a physical map by a next generation sequence approach in barley [34] permitted the assignment of the investigated genes on barley contigs (Table 7). In all cases, the results obtained by genetic mapping were confirmed by the best BLAST-hit, including two locations for different CHS fragments on chromosomes 1H and 6HL. This confirmed previous mapping results, where CHS was placed on chromosome 5 (old nomenclature) equaling chromosome 1H (new nomenclature) [35]. Additional secondary BLAST hits indicated the presence of further gene copies for all tested genes (Table 7).

**Table 7:** Physical mapping of candidate genes

**Gene fragment**	**Best blastN hits**	**Chromosome**	**Score**	**E value**
PAL_1	morex_contig_46437	2HL	1061	0.0
	morex_contig_40780	2HL	841	0.0
	morex_contig_8668	2HL	810	0.0
	morex_contig_103333	2HS	673	0.0
	morex_contig_52512	6HL	664	0.0
	morex_contig_135397	6HL	598	8e-169
	morex_contig_49473	6HL	554	9e-156
	morex_contig_1944918	2HS	533	3e-149
	morex_contig_244188	1H	452	8e-125
	morex_contig_138406	3HS	370	2e-100
	morex_contig_2558942	1H	172	1e-40
PAL_2	morex_contig_46437	2HL	1050	0.0
	morex_contig_8668	2HL	848	0.0
	morex_contig_103333	2HS	722	0.0
	morex_contig_52512	6HL	717	0.0
	morex_contig_135397	6HL	645	0.0
	morex_contig_49473	6HL	587	2e-165
	morex_contig_2558942	1H	513	3e-143
	morex_contig_138406	3HS	497	2e-138
	morex_contig_281235	2HL	468	1e-129
	morex_contig_40780	2HL	410	2e-112
	morex_contig_1586542	2HS	370	2e-100
	morex_contig_1944918	2HS	288	2e-75
	morex_contig_244188	1H	170	4e-40
C4H_1	morex_contig_135422	3HL	877	0.0
C4H_4	morex_contig_135422	3HL	1178	0.0
	morex_contig_57093	7HL	416	6e-114
	morex_contig_54181	3HS	361	1e-97
	morex_contig_1569145	1H	333	6e-89
CHS_1	morex_contig_127876	1H	455	3e-126
	morex_contig_45546	1H	300	1e-79
	morex_contig_140601	2HS	181	8e-44
	morex_contig_65180	2HL	172	4e-41
	morex_contig_48619	2HS	165	6e-39
	morex_contig_359532	1H	154	1e-35
CHS_2	morex_contig_127876	1H	457	7e-127
	morex_contig_45546	1H	277	1e-72
	morex_contig_359532	1H	242	3e-62
	morex_contig_65180	2HL	141	7e-32
	morex_contig_48619	2HS	141	7e-32
	morex_contig_140601	2HS	136	3e-30
CHS_GM290	morex_contig_127876	1H	1150	0.0
	morex_contig_45546	1H	798	0.0
	morex_contig_65180	2HL	605	6e-171
	morex_contig_140601	2HS	578	8e-163
	morex_contig_48619	2HS	533	3e-149
	morex_contig_38618	1H	178	3e-42
	morex_contig_359532	1H	138	2e-30
	morex_contig_37159	6HL	120	7e-25
CHS_GM293	morex_contig_45546	1H	839	0.0
	morex_contig_127876	1H	605	4e-171
	morex_contig_65180	2HL	488	9e-136
	morex_contig_140601	2HS	452	6e-125
	morex_contig_48619	2HS	412	5e-113
	morex_contig_359532	1H	217	2e-54
	morex_contig_96161	-	156	7e-36
	morex_contig_37159	6HL	150	3e-34
	morex_contig_38618	1H	143	4e-32
CHS_GM287	bowman_contig_128263	6HL	571	6e-161
	morex_contig_96161	-	571	6e-161
	morex_contig_42645	4HS	188	7e-46
F3H_1	morex_contig_48553	2HL	495	1e-137
	morex_contig_52807	1H	421	2e-115
	morex_contig_48831	2HL	408	1e-111
	morex_contig_367028	4HL	365	1e-98
	morex_contig_47538	7HS	361	1e-97
	morex_contig_1562556	7HL	318	2e-84
F3H_GM022	morex_contig_48553	2HL	931	0.0
DFR_1	morex_contig_50663	3HL	874	0.0
	morex_contig_90563	6HL	352	5e-95
	morex_contig_77596	6HL	320	3e-85
DFR_4	morex_contig_50663	3HL	1442	0.0
	morex_contig_90563	6HL	875	0.0
	morex_contig_77596	6HL	830	0.0

Blast *N* was used to anchor the genomic *PCR* fragments onto the sequence of barley.

### Association to kernel and malting quality parameters

Barley grain quality parameters are inevitable to evaluate the utility of grains to ensure constant conditions during the malting process. Associations of genetic variation to phenotypic characteristics help to identify molecular markers responsible for good malting quality. They can serve as a selection tool for accelerating breeding processes. The phenotypic variation of different malting and kernel quality parameters were related to structural genetic differences (SNPs, InDels).

For certain candidate genes, the haplotype distribution within the set of 190 European barley cultivars follows their assignment to spring or winter type, or row number. For example, haplotypes DFR_1 and DFR_2 as well as PAL_H1 are mostly represented in two-rowed varieties (Table 2 and 6). Similar results were obtained for *C4H* (Table 3), where haplotype C4H_1 also represents mainly two-rowed varieties. The haplotypes H1, H2 and H3 of *F3H* are mostly found in the spring-pool whereas the winter varieties mainly were assigned to the most abundant haplotype F3H_H1 (Table 5). As a high population structure effect was revealed for these barley populations a correction for population structure within this set of barley accessions is necessary when performing an association study in order to reduce the amount of false positives and negatives [36]).

Three different models were taken into account in order to reveal significant marker-trait associations: principle component analysis (PCA) by using a combination of population structure and kinship, general linear model (GLM) by using population structure and mixed linear model (MLM) by using kinship. Information about population structure was based on a Q5-matrix with random 22 SSR markers. As it was shown in a previous study, multi-allelic SSR markers serve as a more accurate tool to reveal the population structure [36].

Association results significant for all three models are given for four genes, namely *PAL*, *C4H*, *F3H*, *DFR* (Tables 8 and 9). The complete data are represented in the Additional file 8.

**Table 8:** **Significant marker trait associations of haplotypes found in 190 European barley cultivars and selected kernel and malting quality parameters for phenylalanine ammonia**-**lyase** (***PAL***), **cinnamate 4**-**hydroxylase** (***C4H***), **flavanone 3**-**hydroxylase** (***F3H***) **and dihydroflavonol reductase** (***DFR***)

**Haplotype**	**Trait**	**Unit**	**MLM_PCA**			**MLM_QK**				**GLM_Q**		
			**P**	**R**^**2 **^**Model**	**R**^**2 **^**Marker**	**P**		**R**^**2 **^**Model**	**R**^**2 **^**Marker**	**P**	**R**^**2 **^**Model**	**R**^**2 **^**Marker**
PAL_H1	KY	dt/ha	0.04*	0.643	0.018	0.007**		0.709	0.022	0.002**^1^	0.597	0.039
PAL_H2	KF	1-9	0.01*	0.371	0.035	0.005**		0.373	0.040	0.004**	0.370	0.043
	KY	dt/ha	0.039*	0.643	0.018	0.046*		0.699	0.012	0.018*	0.582	0.024
PAL_H3	KF	1-9	0.006**	0.378	0.042	0.016*		0.363	0.030	0.009**	0.361	0.034
C4H_H1	KY	dt/ha	0.007**	0.655	0.030	0.004**^2^		0.711	0.025	0.001***^2^	0.601	0.043
F3H_H1	KRP	%	0.001***^3^	0.605	0.031	0.002**^3^		0.568	0.025	0.002**^3^	0.568	0.025
	KY	dt/ha	0.003**	0.662	0.036	0.014*		0.584	0.026	0.014*	0.584	0.026
F3H_H3	KRP	%	0.006**	0.595	0.021	0.001**^3^		0.572	0.028	0.001***^3^	0.572	0.028
	pH	pH	0.035*	0.375	0.032	0.011*		0.253	0.048	0.011*	0.253	0.048
F3H_H4	FiAt	%	0.002**^3^	0.376	0.070	0.025*		0.090	0.043	0.025*	0.090	0.043
	FEX	%	0.007**	0.522	0.073	0.046*		0.199	0.050	0.046*	0.199	0.050
DFR_H3	SF 2.2-2.5 mm	%	0.033*	0.250	0.028	0.025*		0.271	0.027	0.030*	0.187	0.028
	Brab	HE	0.022*	0.557	0.037	0.002**^3^		0.496	0.063	0.001***^3^	0.214	0.117
	Visc	mPas	0.031*	0.679	0.017	0.004**		0.681	0.027	0.004**	0.494	0.043

Different statistical linear models were considered: 1. Mixed linear model (*MLM*) with principal component analysis (*PCA*), 2. *MLM* with kinship (*K*), 3. General linear model (*GLM*) with population structure (*Q*), significant at *P ≤ 0.05, **P ≤ 0.01, ***P ≤ 0.001 or after Bonferroni correction with ^1^P < 0.0033, ^2^P < 0.005, ^3^P < 0.0025. Trait abbreviations are: *KY* = kernel yield [dt/ha], *KF* = kernel formation [1-9], *SF* = Sieve fraction, *K*_*RP* = kernel raw protein [%], *pH* = *ph*-value, *FiAt* = final attenuation, *FEX* = Fermentable extract, Brab= brabender, Visc = viscosity [mPas].

**Table 9:** **Significant marker trait associations between SNPs and selected kernel and malting quality parameters detected in a set of 190 European barley cultivars for following candidate genes of the phenylpropanoid pathway**: **phenylalanine ammonia**-**lyase** (***PAL***), **cinnamate 4**-**hydroxylase** (***C4H***), **flavanone 3**-**hydroxylase** (***F3H***) **and dihydroflavonol reductase** (***DFR***)

**Marker**	**Trait**	**Unit**	**MLM_PCA**			**MLM_QK**			**GLM_Q**		
			**P**	**R**^**2 **^**Model**	**R**^**2 **^**Marker**	**P**	**R**^**2 **^**Model**	**R**^**2 **^**Marker**	**P**	**R**^**2 **^**Model**	**R**^**2 **^**Marker**
PAL_SNP1	KY	dt/ha	0.041*	0.643	0.018	0.007**	0.709	0.022	0.002**^1^	0.597	0.039
PAL_SNP3	KF	1-9	0.006**	0.373	0.043	0.015*	0.358	0.031	0.009**	0.356	0.036
	Glass	%	0.000***^1^	0.499	0.132	0.000***^1^	0.563	0.079	0.000***^1^	0.271	0.147
PAL_SNP4 + PAL_SNP5	KF	1-9	0.006**	0.378	0.042	0.016*	0.363	0.030	0.009**	0.361	0.034
	Glass	%	0.000***^1^	0.499	0.132	0.000***^1^	0.562	0.079	0.000***^1^	0.271	0.147
C4H_SNP1	KY	dt/ha	0.047*	0.677	0.016	0.010**	0.746	0.018	0.003**	0.645	0.034
C4H_SNP2	Visc	mPas	0.026*	0.680	0.018	0.000***^1^	0.703	0.048	0.000***^1^	0.519	0.068
C4H_SNP4	Visc	mPas	0.032*	0.670	0.018	0.000***^1^	0.694	0.050	0.000***^1^	0.503	0.071
CHS_GM287_SNP1	Visc	mPas	0.001***^2^	0.702	0.041	0.000***^2^	0.726	0.071	0.000***^2^	0.576	0.125
F3H1_SNP38 + F3H_GM022_SNP1	K_RP	%	0.002**	0.601	0.027	0.014*	0.592	0.015	0.010*	0.561	0.018
	KY	dt/ha	0.038*	0.645	0.018	0.039*	0.700	0.013	0.048*	0.575	0.017
F3H_GM022_SNP2	K_RP	%	0.001***^3^	0.606	0.031	0.004**	0.598	0.021	0.003**	0.568	0.024
	KY	dt/ha	0.038*	0.645	0.018	0.022*	0.703	0.016	0.024*	0.580	0.022
F3H_GM022_SNP3	K_RP	%	0.024*	0.586	0.015	0.004**	0.594	0.021	0.003**	0.564	0.024
DFR_SNP4	Brab	HE	0.013*	0.563	0.042	0.010*	0.481	0.044	0.005**	0.183	0.086

Three different statistical models were considered: 1. Mixed linear model (*MLM*) with principal component analysis (*PCA*). 2. *MLM* with kinship (*K)*, 3. General linear model (*GLM*) considering population structure, significant at *P ≤ 0.05, **P ≤ 0.01, ***P ≤ 0.001 or after Bonferroni correction with ^1^P < 0.0025, ^2^P < 0.01, ^3^P < 0.0014. Trait abbreviations are: *KY* = kernel yield [dt/ha], *KF* = kernel formation [1-9], *K*_*RP* = kernel raw protein [%], Brab= brabender, Glass = glassiness, Visc = viscosity [mPas].

Three haplotypes represented by four SNPs of the *PAL* gene were taken into account to reveal their impact on kernel and malting quality parameters. The haplotypes PAL_H2 and PAL_H3 are significant for kernel formation (Table 8). Both haplotypes share the same allele for SNP_1 but differ in SNP3, SNP4 and SNP5, which are individually significant for this trait. SNP1 was found to be significant for kernel yield. This trait is also influenced by haplotypes H1 and H2 that differ in SNP1. The SNPs 3, 4 and 5 were highly significant for glassiness and kernel formation in all three models (Table 9). A high portion of glassy kernels in a malting barley charge leads to an unsatisfactory brewing result. They cause higher turbidity and opal wort, which is not desired. Another important malting parameter is the ‘Brabender’, which serves as an indicator for cytolytic degradation processes in the barley grain. Here, a high value is desirable. Kernel yield (KY) was significantly correlated with SNP1. The allele A coding for adenine in the *P*AL gene is responsible for the significant association of the haplotype PAL_H1 with higher yield (Additional file 8).

All polymorphic sites found for *C4H* were associated with kernel yield and viscosity. The malting parameter viscosity describes the degradation of hemicelluloses catalyzed by endo-β-glucanases. This factor influences the foam stability of the beer and lautering time.

**Figure 7:**
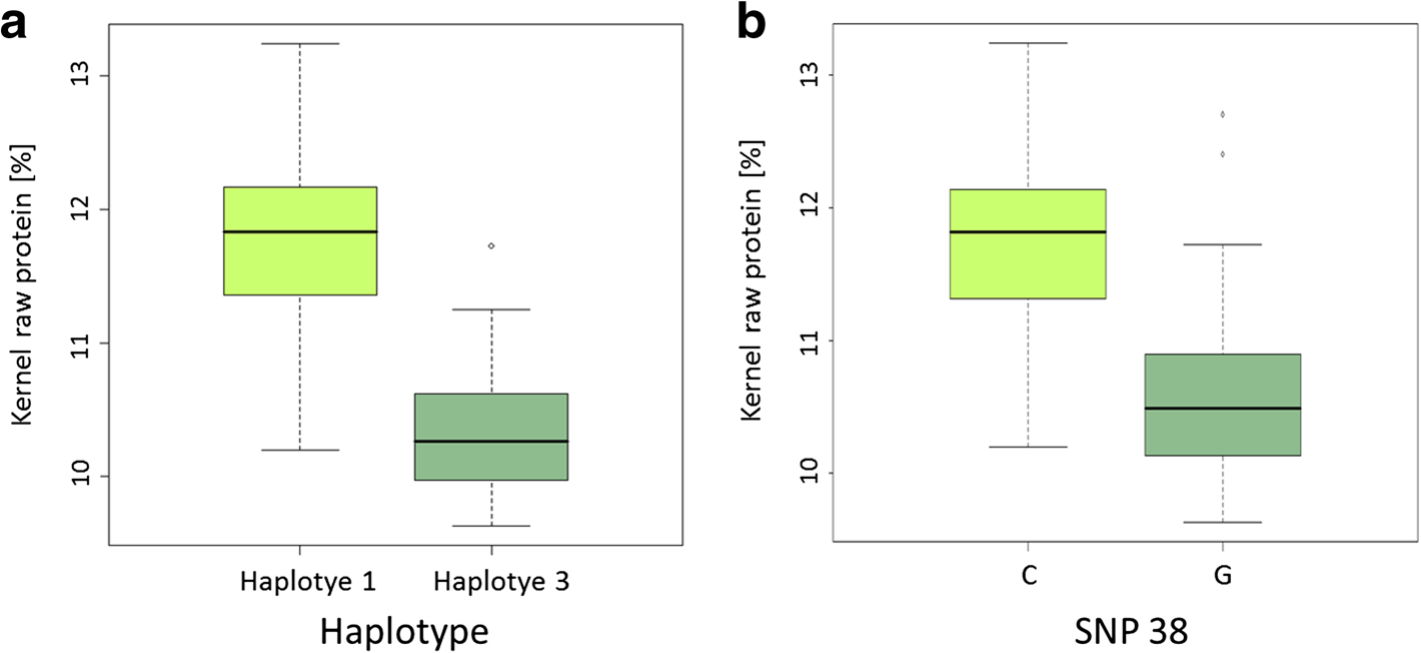
**Level of kernel raw protein assigned to a) *F3H *haplotypes 1 and 3 and b) to the allelic distribution of SNP38 detected in the *F3H *encoding gene.** ┴ = minimum values, ┬ = maximum values, boxes are 0.25 to 0.75 quartiles including ─ = median.

Final attenuation and fermentable extract were significantly associated with the haplotype 4 of *F3H*, which occurs only in two-rowed spring barleys (Table 5). The final attenuation describes the enzymatic activity of amylases and is correlated to dilution properties of the malt extract. A close correlation exists with the malting parameter fermentable extract [37]. Combining SNPs to haplotype patterns can provide more information than single SNPs. This is illustrated by the significant association results of F3H_H4 with these traits, which was not found considering their single SNPs. The haplotypes 1 and 3 of *F3H* showed associations to kernel raw protein (KRP), kernel yield (KY) and pH (Table 8). The haplotype 3 (F3H_H3) is associated to a lower raw kernel protein content (K_RP) and is mainly found in the 2-rowed spring cultivars (Table 5). Differences in raw kernel protein (K_RP) assigned to different SNP alleles (a) or haplotypes (b) for *F3H* are shown in Figure 7. For a good malting and brewing quality, a low raw protein concentration in kernels is desired.

Only one SNP of the *CHS* gene could be converted into a pyrosequencing marker. For this marker trait association (MTA), a high variance was observed for viscosity of the malt extract. Low viscosity values were mainly found in accessions having the favorable C-allele of SNP1, which was true for all investigated 2-rowed spring barleys.

In case of *DFR*, haplotype 3 was found to be significantly associated with sieve fraction (SF), brabender and viscosity. This haplotype is predominantly present in winter cultivars.

In some cases, trait-gene combinations were significant with single SNPs as well as with haplotypes, such as kernel yield and kernel formation with PAL, kernel yield with C4H, kernel raw protein with F3H and Brabender with DFR. Here the evaluation was based on the P-values (Tables 8 and 9), while the R^2^ values explain the percentage of phenotypic variance explained by the model and by the marker. In some cases, the associations of the haplotypes confirm the SNPs, f.e. PAL_SNP1 is specific for PAL_H1 (Table 2) and both were significant for kernel yield (Tables 8 and 9). Another example is the significant association of F3H_H1 with kernel raw protein which is also detected with F3H1_SNP38, a marker specific for F3H_H1 (Table 5).

When Bonferroni correction for multiple testing was applied, the number of significant associations was reduced (Tables 8 and 9). For SNPs, glassiness with PAL and viscosity with CHS remained significant in all three models, while viscosity with C4H was significant with two models. For the haplotypes, kernel raw protein with F3H remained significant in three models, and kernel yield with C4H and Brabender with DFR were significant in two models after correction for multiple testing. By empiric means it cannot be decided which statistic model is the “correct model”, therefore significant associations observed in several statistical models are a good indication for the reliability of a marker trait association.

The comparison of our association results to other malting quality QTL-studies [38,39] was difficult due to the use of different markers in these maps. A QTL for kernel plumpness, malt extract and diastatic power proximal of marker MWG938 on chromosome 1H is in the approximate location of our mapped CHS PCR fragment GM293 [40]. However, we did not discover any marker trait association for this fragment, while our newly detected MTAs were not described before.

While the testing of bi-allelic SNPs only permits the forming of two groups during association analysis, the combination of several SNPs to a haplotype results in a multi-allelic genotypic entity. In both cases, the resulting significance levels were often relatively low. A significant association between a trait and a SNP or haplotype of a gene does not proof the causal functional involvement of a gene in trait expression. The association also could be obtained by linkage disequilibrium present in the genome and also other factors, such as effects of environment play a role. In fact, some of the tested traits, such as color of beer are possibly caused by genes of the phenylpropanoid pathway. Other tested traits like kernel development appear to be unrelated to this pathway. Therefore, the newly developed SNP-markers are related to this specific metabolic pathway described here.

## Conclusions

A high gene-dependent variation of SNP-patterns was found for all candidate genes related to the phenylpropanoid pathway. Combination of SNPs to haplotypes revealed in most cases the divergence of habit (winter and spring varieties) and row number. Most of the developed pyrosequencing markers are applicable for high-throughput genotyping of barley varieties. Several significant associations of the investigated candidate genes with kernel and malting quality traits were detected. Some of the derived SNP-markers can serve as diagnostic tool for marker-assisted selection in breeding programs to achieve new high quality malting varieties.

## Methods

### Plant material and DNA extraction

A set of 16 diverse reference genotypes including the mapping parent Steptoe × Morex was used for resequencing and SNP-detection in five candidate genes from the phenylpropanoid pathway. Additionally, 190 barley varieties of European origin (Additional file 9), consisting of 94 spring and 96 winter types were genotyped with pyrosequencing markers from selected SNPs and InDels. The seeds for growing young plantlets were supplied by various breeding companies or obtained from the Genebank of the Leibniz Institute of Plant Genetics and Crop Plant Research (IPK) at Gatersleben. Harvested leaves from 5 to 6 plants were pooled and the genomic DNA was extracted according to a modified method of Plaschke et al. [41].

### PCR, DNA sequencing and SNP detection

Sequence information (mRNAs, cDNAs or ESTs) for five genes from the phenylpropanoid pathway was obtained from NCBI (http://www.ncbi.nlm.nih.gov/) and are shown in Table 10. All publicly available sequences were aligned with Sequencher^TM^ Vers. 4.06 (Gene Codes Corporation, Ann Arbor, USA). From the consensus sequences of each candidate gene, primers were designed with Primer3 Vers. 0.4.0 [42] and are shown in Additional file 10. The genomic PCR-amplification was performed in 25 μl volume of PCR buffer (0.01 M Tris, 0.05 M KCl, 1.5 mM MgCl_2_, 0.01% gelatine) and contained 100 ng of genomic DNA, 0.2 mM of dCTP, dGTP, dTTP, dATP, 0.2 μM of each primer and 1 U of *Taq* polymerase. After 3 min at 94°C, 45 cycles were performed with 1 min at 94°C, 1 min at 55°C, 2 min at 72°C and a final extension step of 10 min at 72°C. Successfully amplified gene fragments obtained for the reference genotypes were resequenced. All sequences for each gene were aligned and compared to the template reference sequence with the software Sequencher^TM^, and SNP and InDel polymorphisms were revealed. The exact positions of 3’- and 5’-UTR, introns and exons were determined by using GeneSeqer developed by Schlueter et al. [43].

**Table 10:** Investigated genes from the phenylpropanoid pathway

**Candidate gene**	**Accession number (NCBI)**	**Reference**
Phenylalanine ammonia-lyase (*PAL*)	AB367438	Miyashita and Shirako [26]
	X97313	Kervinen et al. [25]
	X99482	Peltonen and Karjalainen [21]
	X99483	
	Z49145	
	Z49146	
	Z49147	
Cinnamate 4-hydroxylase (*C4H*)	AK250541	Sato et al. [27]
	NM_001051180	Rice Annotation Project [42]
	NM_001053349	
	NM_001053354	
	NM_001061725	
Chalcone synthase (*CHS*)	Y09233	Christensen et al. [28]
	U43494	Lee et al. [29]
Flavanone 3-hydroxylase (*F3H*)	X58138	Meldgaard [9]
Dihydoflavonol reductase (*DFR*)	NM_001050192 Hv.23226 (69 ESTs)	Rice Annotation Project [42]

NCBI accession numbers for the template sequences used for the genomic primer design are given.

### Marker development

Pyrosequencing assays were developed for high-throughput genotyping of the 190 cultivars and the segregating mapping populations. The primer combinations were designed with the PSQ Assay Design Software Version 1.0.6) provided by Biotage (Uppsala, Sweden) and are summarized in Additional file 10. The PCR-reactions with labeled biotin primers were performed in a 35 μl reaction volume with an annealing temperature of 58°C. All pyrosequencing assays were carried out according to the manufacturer’s standard protocols using a pyrosequencer PSQ HS 96 from Biotage AB (Uppsala, Sweden). The analyzed SNP- and InDel-data were scored with the manufacturer’s software.

CAPS marker development was performed using the program SNP2CAPS by Thiel et al. [44]. The digestions were carried out in 20 μl reaction volumes with 15 μl of PCR amplification product, 2 μl 10× buffer and 2 U restriction enzyme. All used restriction enzymes are summarized in Additional file 11.

### Mapping

All SNP-markers found to be polymorphic either between the mapping parents Steptoe x Morex or Morex x Barke were tested on each double haploid (DH) mapping population. The Steptoe x Morex population consisted of a set of 77 double haploid lines. Linkage maps were constructed using the software MapMaker 2.0 [45]. Genetic distances were calculated by applying the Kosambi function [46]. The segregating population of Morex x Barke was kindly provided by Nils Stein (Leibniz Institute of Plant Genetics and Crop Plant Research, Gatersleben, Germany) and consisted of 93 DH lines. Calculation was performed using JoinMap Software [47] based on a set of DArT and SNP markers [31,32].

For anchoring the investigated genes on the physical map, a blastN of the PCR-derived sequences was conducted by using the IPK BLAST server (http://webblast.ipk-gatersleben.de/barley/) on the database assembly_WGSMorex and in case of fragment GM287 on assembly_WGSBowman.

### Association studies

Association studies were performed using the TASSEL software, vers. 2.1.1 developed by Bradbury et al. [48]. Three linear models were applied taking either principal components (PCA), kinship (K) and/or population structure (Q) into account.

The population structure applied in the GLM was implemented as a Q-matrix reflecting the relative assignments of 22 random SSRs [49] to five subgroups. The genetic relationship or kinship was determined by SPAGeDi [50] applying the Ritland [51] coefficient using 22 random SSR markers. The kinship information was implemented in the MLM [36]. All calculations were performed as described previously [21]. The threshold for Bonferroni correction for multiple testing was calculated for each investigated gene separately by dividing P<0.01 with the number of SNPs or haplotypes of the respective gene.

All investigations were carried out using SNP and InDel markers or by combining all marker data in haplotypes for each gene applying on a set of 190 European barley accessions that reflect 96 winter and 94 spring cultivars. Phenotypic values considered for this candidate gene association approach were derived from the database MetaBrew [52]. The following grain and malting quality parameters were considered: kernel yield [dt/ha], kernel formation [1-9], thousand grain weight [g], kernel raw protein [%], raw protein in malt [%], pH, sieve fraction [%], hectolitre weight [kg], brabender [HE], diastatic power [WK], final attenuation [%], fermentable extract [%], malt extract [%], color [EBC], friability [%], glassiness [%], soluble nitrogen [mg/100g dry malt], malting quality index [MQI], malt extract [%], viscosity [mPas], saccharification VZ45 [%]. These data were published from various German state trials in different years, at different locations including variable sets of varieties per trial and year. Each trait was covered by 2–103 single entries per variety (Additional file 12). Outliers deviating more than 20% from the mean were discarded. Mean values for each trait/variety combination were calculated over all available single entries (Additional file 13). Only mean values based on at least 20 single entries out of the total varietal set were taken into account. Due to availability of phenotypic data only up to 185 varieties were used for association analysis of the individual traits (Additional file 13).

## Competing interests

The authors declare that they have no competing interests.

## Authors’ contributions

IEM and MSR conceived and supervised the project. MP conducted the scientific research, performed data analysis and drafted the manuscript. SW helped with data analysis. All authors read and approved the manuscript.

## Supplementary Material

Additional file 1 **SNPs detected within 16 reference genotypes for the phenylalanine ammonia-lyase (*PAL*) gene fragment PAL_1.** Click here for file

Additional file 2 **SNPs detected within 16 reference genotypes for the phenylalanine ammonia-lyase (*PAL*) gene fragment PAL_2.** Click here for file

Additional file 3 **Genetic structure of the resequenced fragments C4H_1 and C4H_4 from the cinnamate 4-hydroxylase (*C4H*) encoding gene.** Double lines indicate UTR regions, single lines indicate no sequenced regions. Violet – CAPS marker and high-throughput SNP marker, green – high-throughput SNP marker. Click here for file

Additional file 4 **Positioning of the chalcone synthase (*CHS*) resequenced gene fragments GM_293 and GM_290, CHS_1, CHS_2 and CHS_3 in relation to cDNA Y09233.** Polymorphisms were only detected in the large fragments GM_290 and GM_293. Green – high-throughput SNP marker. Click here for file

Additional file 5 **Detected SNPs and their resulting haplotypes within 16 reference genotypes for the chalcone synthase (*CHS*) gene fragment GM_293.** Click here for file

Additional file 6 **Detected SNPs within 16 reference genotypes for the chalcone synthase (*CHS*) gene fragment GM_287.** Four different haplotypes were identified. Click here for file

Additional file 7 **Genetic structure of dihydoflavonol reductase (*DFR*) according to Kristiansen and Rohde **[30]**.** Primer development for resequencing was performed using sequence information of EST contig Hv.23226. Light grey boxes represent exons and double lines between exons indicate the introns. UTRs are also marked by doubled lines. Green – high-throughput SNP marker, violet – CAPS marker and high-throughput SNP marker. Click here for file

Additional file 8 **Marker-trait-associations of SNP polymorphisms and haplotypes found in 190 European barley cultivars and selected kernel and malting quality parameters for cinnamate 4-hydroxylase (*C4H*), flavanone 3-hydroxylase (*F3H*), dihydoflavonol reductase (*DFR*), chalcon synthase (*CHS*) and phenylalanine ammonia-lyase (*PAL*).** Considered statistical models: 1. Mixed linear model (MLM) with principal component analysis (PCA), 2. MLM with kinship, 3. General linear model (GLM) with population structure. Significant at * p < 0.05, ** p < 0.01,*** p < 0.001, n.s. not significant. Click here for file

Additional file 9 List of investigated 190 barley varieties. Click here for file

Additional file 10 List of primers used for PCR amplification and sequencing. Click here for file

Additional file 11 Used restriction enzymes for CAPS marker development and reaction conditions. Click here for file

Additional file 12 Trait statistics of single phenotypic values across 185 varieties (before elimination of outliers) for 22 traits. Click here for file

Additional file 13 Phenotypic data used for association analysis (mean values for each trait/variety combination after elimination of outliers). Click here for file
