# Supplementary material for: Development of SNP markers for genes of the phenylpropanoid pathway and their association to kernel and malting traits in barley
Source: BMC Genet. 2013 Oct 2;14:97. doi: 10.1186/1471-2156-14-97 (PMC3852699; doi:10.1186/1471-2156-14-97)
Supplement: Additional file 1 — SNPs detected within 16 reference genotypes for the phenylalanine ammonia-lyase (PAL) gene fragment PAL_1. [file 1471-2156-14-97-S1.docx]

**Additional file 1** – SNPs detected within 16 reference genotypes for the phenylalanine ammonia-lyase (*PAL*) gene fragment PAL_1.

| **bp-position** | 33 | 45 | 306 | 537 | 543 |  |
| --- | --- | --- | --- | --- | --- | --- |
| **SNP** | SNP1 | SNP2 | SNP3 | SNP4 | SNP5 |  |
| **Code** | CCC=Pro | TCC=Ser | CTC=Leu | CGA=Arg | GCG=Ala |  |
|  | CCA=Pro | TCT=Ser | CTT=Leu | CGT=Arg | GCA=Ala | **Haplotype** |
| Steptoe | c | c | T | T | A | PAL_1_H3 |
| Morex | C | C | C | A | G | PAL_1_H2 |
| Igri | a | T | C | A | G | PAL_1_H1 |
| Franka | c | c | C | A | G | PAL_1_H2 |
| OWB-dom | c | T | C | A | G | PAL_1_H4 |
| OWB-rec | c | c | C | A | G | PAL_1_H2 |
| Brenda | a | T | C | A | G | PAL_1_H1 |
| *H. sp.* 584 | a | t | C | A | G | PAL_1_H1 |
| Steina | a | T | C | A | G | PAL_1_H1 |
| Alexis | a | T | C | A | G | PAL_1_H1 |
| Steffi | A | T | C | A | G | PAL_1_H1 |
| Marthe | A | T | C | A | G | PAL_1_H1 |
| Tiffany | c | c | T | T | A | PAL_1_H3 |
| Vanessa | a | T | C | A | G | PAL_1_H1 |
| Lomerit | c | c | T | T | A | PAL_1_H3 |
| Verena | c | c | C | A | G | PAL_1_H2 |
