# Supplementary material for: Development of SNP markers for genes of the phenylpropanoid pathway and their association to kernel and malting traits in barley
Source: BMC Genet. 2013 Oct 2;14:97. doi: 10.1186/1471-2156-14-97 (PMC3852699; doi:10.1186/1471-2156-14-97)
Supplement: Additional file 2 — SNPs detected within 16 reference genotypes for the phenylalanine ammonia-lyase (PAL) gene fragment PAL_2. [file 1471-2156-14-97-S2.docx]

Additional file 2 – SNPs detected within 16 reference genotypes for the phenylalanine ammonia-lyase (*PAL*) gene fragment PAL_2.

| **bp-Position** | 66 | 90 | 93 | 114 | 117 | 159 | 162 | 177 | 216 | 222 | 243 | 267 | 282 | 288 | 300 |
| --- | --- | --- | --- | --- | --- | --- | --- | --- | --- | --- | --- | --- | --- | --- | --- |
| **SNP** | SNP1 | SNP2 | SNP3 | SNP4 | SNP5 | SNP6 | SNP7 | SNP8 | SNP9 | SNP10 | SNP11 | SNP12 | SNP13 | SNP14 | SNP15 |
| **Code** | ATA=Ile | ATG=Met | CTT=Leu | CTC=Leu | GAC=Asp | ACG=Thr | TCG=Ser | GGA=Gly | ATT=Ile | CGA=Arg | GAC=Asp | CGT=Arg | CAT=His | GGC=Gly | GGC=Gly |
|  | ATC=Ile | ATA=Ile | CTC=Leu | CTT=Leu | GAT=Asp | ACA=Thr | TCA=Ser | GGC=Gly | ATC=Ile | CGG=Arg | GAT=Asp | CGG=Arg | CAC=His | GGT=Gly | GGT=Gly |
| Steptoe | C | A | C | T | T | A | A | C | C | G | T | G | C | T | C |
| Morex | A | G | T | C | C | G | G | C | T | A | C | T | T | C | T |
| Igri | A | G | T | C | C | G | G | A | T | A | C | T | T | C | C |
| Franka | A | G | T | C | C | G | G | C | T | A | C | T | T | C | C |
| OWB-dom | A | G | T | C | C | - | G | A | T | A | C | T | - | C | C |
| OWB-rec | A | G | T | C | C | G | G | C | T | A | C | T | T | C | T |
| Brenda | A | G | T | C | C | G | G | A | T | A | C | T | T | C | C |
| *H. sp.* 584 | A | G | T | C | C | G | G | A | T | A | C | T | T | C | C |
| Steina | A | G | T | C | C | G | G | A | T | A | C | T | T | C | C |
| Alexis | A | G | T | C | C | G | G | A | T | A | C | T | T | C | C |
| Steffi | A | G | T | C | C | G | G | A | T | A | C | T | T | C | C |
| Marthe | A | G | T | C | C | G | G | A | T | A | C | T | T | C | C |
| Tiffany | C | A | C | T | T | A | A | C | C | G | T | G | C | T | C |
| Vanessa | A | G | T | C | C | G | G | A | T | A | C | T | T | C | C |
| Lomerit | C | A | C | T | T | A | A | C | C | G | T | G | C | T | C |
| Verena | A | G | T | C | C | G | G | C | T | A | C | T | T | C | C |

| **bp-Position** | 306 | 318 | 324 | 330 | 351 | 360 | 382 | 384 | 394 | 411 | 414 | 423 | 426 | 429 | 495 | 498 |
| --- | --- | --- | --- | --- | --- | --- | --- | --- | --- | --- | --- | --- | --- | --- | --- | --- |
| **SNP** | SNP16 | SNP17 | SNP18 | SNP19 | SNP20 | SNP21 | SNP22 | SNP23 | SNP24 | SNP25 | SNP26 | SNP27 | SNP28 | SNP29 | SNP30 | SNP31 |
| **Code** | CCA=Pro | TCT=Ser | GAC=Asp | ACC=Thr | ATT=Ile | CTC=Leu | TTG=Leu | CTA=Leu | CTC=Leu | CTA=Leu | CCT=Pro | CTT=Leu | TCC=Ser | GGT=Gly | TCT=Ser | GAG=Glu |
|  | CCC=Pro | TCC=Ser | GAT=Asp | ACG=Thr | ATC=Ile | CTT=Leu |  |  | TTC=Phe | CTG=Leu | CCC=Pro | CTC=Leu | TCT=Ser | GGC=Gly | TCC=Ser | GAA=Glu |
| Steptoe | C | C | T | G | C | T | C | A | T | A | C | C | T | C | C | A |
| Morex | C | C | C | C | T | C | T | G | C | G | T | T | C | T | T | G |
| Igri | A | T | C | C | T | C | T | G | C | G | T | T | C | T | T | G |
| Franka | C | C | C | C | T | C | T | G | - | - | T | T | - | T | T | G |
| OWB-dom | - | - | C | C | - | C | - | G | - | - | T | - | C | - | - | G |
| OWB-rec | C | C | C | C | T | C | T | G | C | A | T | T | C | T | T | G |
| Brenda | A | T | C | C | T | C | T | G | C | A | T | T | C | T | T | G |
| *H. sp.* 584 | A | T | C | C | T | C | T | G | C | A | T | T | C | T | T | G |
| Steina | A | T | C | C | T | C | T | G | C | A | T | T | C | T | T | G |
| Alexis | A | - | C | C | T | C | T | G | C | A | T | - | C | T | T | G |
| Steffi | A | T | C | C | T | C | T | G | C | A | T | T | C | T | T | G |
| Marthe | A | T | C | C | T | C | T | G | C | A | T | T | C | T | T | G |
| Tiffany | C | C | T | G | C | T | C | A | T | G | C | C | T | C | C | A |
| Vanessa | A | T | C | C | T | C | T | G | C | A | T | T | C | T | T | G |
| Lomerit | C | C | T | G | C | T | C | A | T | G | C | C | T | C | C | A |
| Verena | C | C | C | C | T | C | T | G | T | - | T | T | C | T | T | G |

| **bp-Position** | 501 | 504 | 519 | 522 |  |
| --- | --- | --- | --- | --- | --- |
| **SNP** | SNP32 | SNP33 | SNP34 | SNP35 |  |
| **Code** | CTC=Leu | CAG=Gln | CCT=Pro | GTG=Val |  |
|  | CTT=Leu | CAA=Gln | CCG=Pro | GTT=Val | Haplotype |
| Steptoe | T | A | G | T | H4 |
| Morex | C | G | T | G | H5 |
| Igri | C | G | T | G | H6 |
| Franka | C | G | T | G | - |
| OWB-dom | C | G | T | G | - |
| OWB-rec | C | G | T | G | H7 |
| Brenda | C | G | T | G | H1 |
| *H. sp.* 584 | C | G | T | G | H1 |
| Steina | C | G | T | G | H1 |
| Alexis | C | G | T | G | - |
| Steffi | C | G | T | G | H1 |
| Marthe | C | G | T | G | H1 |
| Tiffany | T | A | G | T | H3 |
| Vanessa | C | G | T | G | H1 |
| Lomerit | T | A | G | T | H3 |
| Verena | C | G | T | G | H2 |
