# Supplementary material for: Development of SNP markers for genes of the phenylpropanoid pathway and their association to kernel and malting traits in barley
Source: BMC Genet. 2013 Oct 2;14:97. doi: 10.1186/1471-2156-14-97 (PMC3852699; doi:10.1186/1471-2156-14-97)
Supplement: Additional file 3 — Genetic structure of the resequenced fragments C4H_1 and C4H_4 from the cinnamate 4-hydroxylase (C4H) encoding gene. Double lines indicate UTR regions, single lines indicate no sequenced regions. Violet – CAPS marker and high-throughput SNP marker, green – high-throughput SNP marker. [file 1471-2156-14-97-S3.docx]

Additional file 3 – Genetic structure of resequenced fragments C4H_1 and C4H_4 from the cinnamate 4-hydroxylase (*C4H*) encoding gene. Double lines indicate UTR regions of the gene, single lines indicate non sequenced regions. Violet – CAPS marker and high-throughput SNP marker, green – high-throughput SNP marker .
