# Supplementary material for: Development of SNP markers for genes of the phenylpropanoid pathway and their association to kernel and malting traits in barley
Source: BMC Genet. 2013 Oct 2;14:97. doi: 10.1186/1471-2156-14-97 (PMC3852699; doi:10.1186/1471-2156-14-97)
Supplement: Additional file 4 — Positioning of the chalcone synthase (CHS) resequenced gene fragments GM_293 and GM_290, CHS_1, CHS_2 and CHS_3 in relation to cDNA Y09233. Polymorphisms were only detected in the large fragments GM_290 and GM_293. Green – high-throughput SNP marker. [file 1471-2156-14-97-S4.docx]

Additional file 4 – Positioning of chalcone synthase (*CHS*) resequenced gene fragments GM_293 and GM_290, CHS_1, CHS_2 and CHS_3 in relation to cDNA Y09233. Polymophisms were only detected in the enlarged fragments GM_290 and GM_293. Green – high-throughput SNP marker .
