# Supplementary material for: Development of SNP markers for genes of the phenylpropanoid pathway and their association to kernel and malting traits in barley
Source: BMC Genet. 2013 Oct 2;14:97. doi: 10.1186/1471-2156-14-97 (PMC3852699; doi:10.1186/1471-2156-14-97)
Supplement: Additional file 5 — Detected SNPs and their resulting haplotypes within 16 reference genotypes for the chalcone synthase (CHS) gene fragment GM_293. [file 1471-2156-14-97-S5.docx]

Additional file 5 – Detected SNPs and their resulting haplotypes within 16 reference genotypes for the chalcone synthase (*CHS*) gene fragment GM_293.

| **bp-Position** | 86 | 88 | 110 | 113 | 122 | 140 | 152 | 164 | 200 | 203 | 269 | 305 | 334 | 335 |  |
| --- | --- | --- | --- | --- | --- | --- | --- | --- | --- | --- | --- | --- | --- | --- | --- |
| **SNP** | SNP1 | SNP2 | SNP3 | GTG=Val | SNP5 | SNP6 | SNP7 | SNP8 | SNP9 | SNP10 | SNP11 | SNP12 | SNP13 | SNP14 |  |
| **Code** | ACT=Thr | AGG=Arg | ACC=Thr | GTA=Val | CTC=Leu | GGC=Gly | GGT=Gly | CTC=Leu | GGT=Gly | GCA=Ala | CTT=Leu | GGC=Gly | C**T**C=Leu | CT**C**=Leu |  |
|  | ACC=Thr | AAG=Lys | ACGThr | GTT=Val | CTG=Leu | GGG=Gly | GGC=Gly | CTT=Leu | GGC=Gly | GCT=Ala | CTC=Leu | GGA=Gly | C**C**T=Pro | CC**T**=Pro | **Haplotyp** |
| Steptoe | C | A | G | G | G | G | C | T | C | T | C | C | C | T | GM293_H4 |
| Morex | T | G | C | A | C | C | T | C | T | A | T | C | T | C | GM293_H1 |
| Igri | T | G | C | - | C | C | T | C | T | A | T | C | T | C | - |
| Franka | T | G | C | T | C | C | T | C | T | A | T | C | T | C | GM293_H3 |
| OWB-dom | T | G | C | T | C | C | T | C | T | A | T | C | T | C | GM293_H3 |
| OWB-rec | T | G | C | T | C | C | T | C | T | A | T | A | T | C | GM293_H2 |
| Brenda | T | G | C | T | C | C | T | C | T | A | T | A | T | C | GM293_H2 |
| H. sp. 584 | T | G | C | T | C | C | T | C | T | A | T | A | T | C | GM293_H2 |
| Steina | T | G | C | A | C | C | T | C | T | A | T | C | T | C | GM293_H1 |
| Alexis | T | G | C | A | C | C | T | C | T | A | T | C | T | C | GM293_H1 |
| Steffi | T | G | C | A | C | C | T | C | T | A | T | C | T | C | GM293_H1 |
| Marthe | T | G | C | - | C | C | T | C | T | A | T | C | T | C | - |
| Tiffany | T | G | C | A | C | C | T | C | T | A | T | C | T | C | GM293_H1 |
| Vanessa | T | G | C | A | C | C | T | C | T | A | T | C | T | C | GM293_H1 |
| Lomerit | T | G | C | A | C | C | T | C | T | A | T | C | T | C | GM293_H1 |
| Verena | T | G | C | A | C | C | T | C | T | A | T | C | T | C | GM293_H1 |

| **bp-Position** | 359 | 414 | 416 |  |
| --- | --- | --- | --- | --- |
| **SNP** | SNP15 | SNP16 | SNP17 |  |
| **Code** | CTC=Leu | AAT=Asn | AAT=Asn |  |
|  | CTG=Leu | GAC=Asp | GAC=Asp | **Haplotype** |
| Steptoe | G | G | C | GM293_H4 |
| Morex | C | A | T | GM293_H1 |
| Igri | C | A | T | - |
| Franka | C | A | T | GM293_H3 |
| OWB-dom | C | A | T | GM293_H3 |
| OWB-rec | C | A | T | GM293_H2 |
| Brenda | C | A | T | GM293_H2 |
| H. sp. 584 | C | A | T | GM293_H2 |
| Steina | C | A | T | GM293_H1 |
| Alexis | C | A | T | GM293_H1 |
| Steffi | C | A | T | GM293_H1 |
| Marthe | C | A | T | - |
| Tiffany | C | A | T | GM293_H1 |
| Vanessa | C | A | T | GM293_H1 |
| Lomerit | C | A | T | GM293_H1 |
| Verena | C | A | T | GM293_H1 |
