# Supplementary material for: Development of SNP markers for genes of the phenylpropanoid pathway and their association to kernel and malting traits in barley
Source: BMC Genet. 2013 Oct 2;14:97. doi: 10.1186/1471-2156-14-97 (PMC3852699; doi:10.1186/1471-2156-14-97)
Supplement: Additional file 6 — Detected SNPs within 16 reference genotypes for the chalcone synthase (CHS) gene fragment GM_287. Four different haplotypes were identified. [file 1471-2156-14-97-S6.docx]

Additional file 6 – Detected SNPs within 16 reference genotypes for the chalcone synthase (*CHS*) gene fragment GM_287. Four different haplotypes were identified

| **Bp-Position** | 136 | 149 | 255 | 279 | 280 | 281 | 282 | 283 | 284 | 285 | 286 |  |
| --- | --- | --- | --- | --- | --- | --- | --- | --- | --- | --- | --- | --- |
| **SNP** | SNP1 | SNP2 | SNP3 | SNP4 | SNP5 | SNP6 | SNP7 | SNP8 | SNP9 | SNP10 | SNP11 |  |
| **Code** | CGT=Arg | GTG=Val | C/T | A/C | T/C | A/G | G/T | T/C | T/A | C/G | T/C |  |
|  | TGT=Cys | GCG=Ala |  |  |  |  |  |  |  |  |  | **Haplotype** |
| Steptoe | T | T | C | a | t | a | g | t | t | c | t | GM287_H2 |
| Morex | c | t | c | - | - | - | - | - | - | -. | -. | - |
| Igri | C | T | C | a | t | a | t | t | t | c | c | GM287_H1 |
| Franka | T | T | C | a | t | a | g | t | t | c | t | GM287_H2 |
| OWB-dom | C | C | T | C | C | G | T | C | A | G | - | GM287_H3 |
| OWB-rec | T | T | C | a | t | a | g | t | t | c | t | GM287_H2 |
| Brenda | C | T | C | a | t | a | t | t | t | c | c | GM287_H1 |
| H. sp. 584 | C | T | C | a | t | a | t | t | t | c | c | GM287_H1 |
| Steina | C | T | C | a | t | a | t | t | t | c | c | GM287_H1 |
| Alexis | C | T | C | a | t | a | t | t | t | c | c | GM287_H1 |
| Steffi | C | T | C | a | t | a | t | t | t | c | c | GM287_H1 |
| Marthe | C | T | C | a | t | a | t | t | t | c | c | GM287_H1 |
| Tiffany | c | t | c | a | t | a | t | t | t | c | c | GM287_H1 |
| Vanessa | C | T | C | a | t | a | t | t | t | c | c | GM287_H1 |
| Lomerit | C | T | C | a | t | a | g | t | t | c | c | GM287_H4 |
| Verena | C | T | C | a | t | a | t | t | t | c | c | GM287_H1 |
