# Supplementary material for: Development of SNP markers for genes of the phenylpropanoid pathway and their association to kernel and malting traits in barley
Source: BMC Genet. 2013 Oct 2;14:97. doi: 10.1186/1471-2156-14-97 (PMC3852699; doi:10.1186/1471-2156-14-97)

**Additional file 7** – Genetic structure of dihydoflavonol reductase (*DFR*) according to Kristiansen and Rohde, 1991 [[30](#_ENREF_30)]. Primer development for resequencing was performed using sequence information of EST contig Hv.23226. Light grey boxes represent exons and double lines between exons indicate the introns. UTRs are also marked by doubled lines. Green – high-throughput SNP marker, violet – CAPS marker and high-throughput SNP marker.


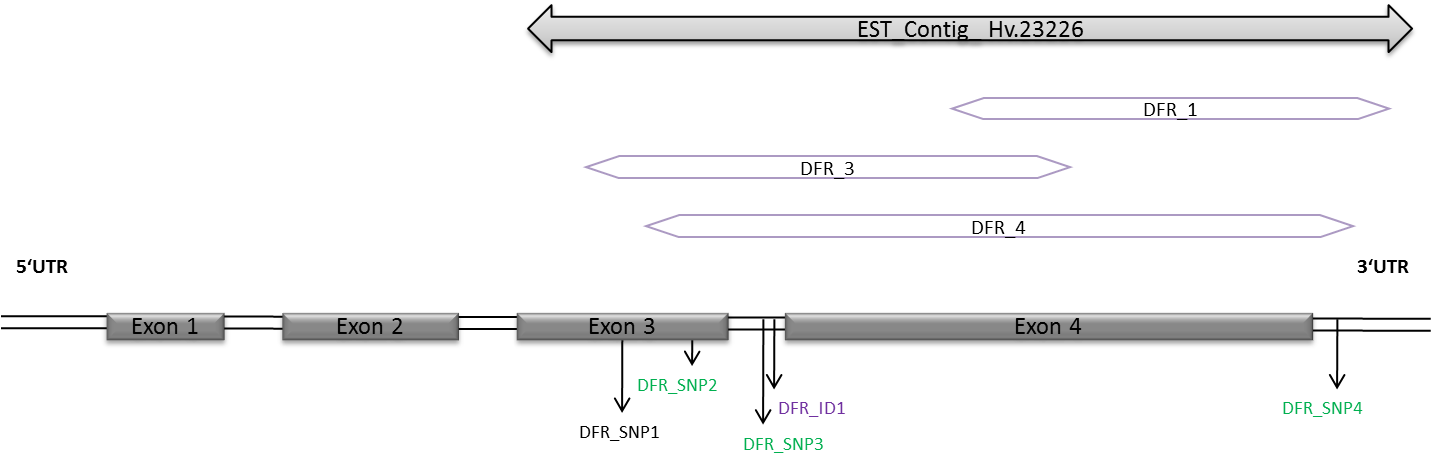

Supplement: Additional file 7 — Genetic structure of dihydoflavonol reductase (DFR) according to Kristiansen and Rohde [30]. Primer development for resequencing was performed using sequence information of EST contig Hv.23226. Light grey boxes represent exons and double lines between exons indicate the introns. UTRs are also marked by doubled lines. Green – high-throughput SNP marker, violet – CAPS marker and high-throughput SNP marker. [file 1471-2156-14-97-S7.docx]
