# Supplementary material for: Development of SNP markers for genes of the phenylpropanoid pathway and their association to kernel and malting traits in barley
Source: BMC Genet. 2013 Oct 2;14:97. doi: 10.1186/1471-2156-14-97 (PMC3852699; doi:10.1186/1471-2156-14-97)
Supplement: Additional file 10 — List of primers used for PCR amplification and sequencing. [file 1471-2156-14-97-S10.docx]

Additional file 10: List of primers used for PCR amplification and sequencing

| **Purpose** | **Primer name and direction** | **Primer sequence 5’…3’** |
| --- | --- | --- |
| **Phenylalanine ammonia-lyase (*PAL*)** | | |
| PCR amplification and sequencing | PAL1_F | GCTATCCATGGTGGCAACTT |
|  | PAL1_R | CACGCATCTTCTGCATGAGT |
|  | PAL2_F | CACTTGACCCACAAGCTGAA |
|  | PAL2_R | TCTTCCTGGAGGAGATGAGG |
| PAL1_SNP1 detection by pyrosequencing | FP_PAL1_SNP1 | CTATCCATGGTGGCAACTTCC |
|  | BRP_PAL1_SNP1 | AGAACTGGAGCTCAGAGCAGTACG |
|  | SFP_PAL1_SNP1 | ACTTCCAGGGCACAC |
| PAL1_SNP3 detection by pyrosequencing | FP_PAL1_SNP3 | TTCTTGGGCAACCCTGTG |
|  | BRP_PAL1_SNP3 | GGTGGCGGAGGTCGATAG |
|  | SFP_PAL1_SNP3 | TCAATTCTCTTGGCCT |
| PAL1_SNP4+5 detection by pyrosequencing | BFP_PAL1_SNP4+5 | GTGTGAAGACGGTGGCTAGGA |
|  | RP_PAL1_SNP4+5 | GTAGTTGGCGCTGCAGGG |
|  | SRP_PAL1_SNP4+5 | CGGCGTAGGCGAACA |
|  |  |  |
| **Cinnamate 4-hydroxylase (*C4H*)** | | |
| PCR amplification and sequencing | C4H1_F | CATCTTGAAGCACTCCACCA |
|  | C4H1_R | GCAATTTGCATTGCAGAAAA |
|  | C4H4_F | CTTCGTCTTCGTGGAGAAGC |
|  | C4H4_R | GGGATGAAGTCCCCGTAGTT |
| C4H1_SNP1 detection by pyrosequencing | FP_C4H1_SNP1 | GATTATGATTGTTCGCTCGTCCTA |
|  | BRP_C4H1_SNP1 | GACATCTCTTCAGCTCCTCTGTTT |
|  | SFP_C4H1_SNP1 | ATTATTATAGTATCTGTCACAGAGT |
| C4H1_SNP2 detection by pyrosequencing | BFP_C4H1_SNP2 | AAGAAACAGAGGAGCTGAAGAGAT |
|  | RP_C4H1_SNP2 | AATTTATCGCAGCATCAGGAGT |
|  | SRP_C4H1_SNP2 | GTCTTTTCCTCCCCG |
| C4H1_SNP3 detection by pyrosequencing | FP_C4H1_SNP3 | CTCCTGATGCTGCGATAAATTAAG |
|  | BRP_C4H1_SNP3 | TCCACACAAGTGATCGTTACAA |
|  | SFP_C4H1_SNP3 | TGCTGCGATAAATTAAGA |
| C4H1_SNP4 detection by pyrosequencing | FP_C4H1_SNP4 | CCCTCCATTTGTTTCTTGTATTCT |
|  | BRP_C4H1_SNP4 | AGCTCCACACAAGTGATCGTTAC |
|  | SFP_C4H1_SNP4 | TTGTTTCTTGTATTCTACTCG |
|  |  |  |
| **Chalcone synthase (*CHS*)** | | |
| PCR amplification and sequencing | GM0287_L | GCAATCTTGGACAGTGTGGA |
|  | GM0287_R | GCTATGCCAGGGCATTTGTA |
|  | GM0290_L | GATCACCCACCTCGTGTTCT |
|  | GM0290_R | GAGTACACTTGCGCTGGACA |
|  | GM0293_L | GGGGCCAGCCATTGTCCA |
|  | GM0293_R | GAGTCCCGGCACGTCCTTGAGAA |
|  | CHS_1_F | CGGTGAGGTTGAAGGAAGTG |
|  | CHS_1_R | CGCAGATCTTGGGGTTCTTA |
|  | CHS_2_F | CAGGCGACCTACCCAGACTA |
|  | CHS_2_R | CCACTCCTTGATTGCCTTCT |
|  | CHS_3_F | CCACTCCTTGATTGCCTTCT |
|  | CHS_3_R | CGGTGAGGTTGAAGGAAGTG |
| SNP1detection of CHS_GM287 by pyrosequencing | BFP_GM287_SNP1 | GCCGACAGTCATCTTCGTCCT |
|  | RP_GM287_SNP2 | TACCAAGTGGCACGCAACAG |
|  | SRP_GM287_SNP3 | CACCCCCCACATGCT |
| **Flavanon 3-hydroxylase (*F3H*)** | | |
| PCR amplification and sequencing | F3H_1_F | AGGACTGGGGCATCTTCC |
|  | F3H_1_R | ATCTCCCTCCAGTCCTGCAC |
|  | GM022_L | CGGCGCATTCGTCGTCAACCTC |
|  | GM022_R | GAATCAATCCCACGCATCCATC AA |
| SNP36-39 detection of F3H_1 by pyrosequencing | FP_F3H1_SNP36-39 | CGGAGGGAGTATCTATCAGCA |
|  | BRP_F3H1_SNP36-39 | TTCCCTTTCAGTCCTGCACC |
|  | SFP_F3H1_SNP36-39 | AAGATGAAATTGACCCA |
| ID4 detection of F3H_1 by pyrosequencing | BFP_F3H1_ID4 | GAACGGAGGGAGTATCTATCAGC |
|  | RP_F3H1_ID5 | TATTCCCTTTCAGTCCTGCACC |
|  | SRP_F3H1_ID4 | CATGGGTCAATTTCATCT |
| SNP1 detection of GM022 by pyrosequencing | BFP_GM022_SNP1 | AGTACCCCCTTCATCTTCTTCCAT |
|  | RP_GM022_SNP1 | CCTGCCGTTGCTCATGAA |
|  | SRP_GM022_SNP1 | TGTTCAATTGGTGTTCG |
| SNP2 detection of GM022 by pyrosequencing | BFP_GM022_SNP2 | GGCGCATTTGTCGTCAACC |
|  | RP_GM022_SNP2 | CCGGGTTCTGGAACGTCG |
|  | SRP_GM022_SNP2 | TGAACTGCATGCACATAGC |
| SNP3 detection of GM022 by pyrosequencing | FP_GM022_SNP3 | ATGTACCGCCGCAAGATG |
|  | BRP_GM022_SNP3 | GCCGGAAGGATCTAGGCAA |
|  | SFP_GM022_SNP3 | AAGATGGAGCGCGAC |
| **dihydroflavonol reductase (*DFR*)** | | |
| PCR amplification and sequencing | DFR_1_F | GCCCACTACTCGATCCTGAA |
|  | DFR_1_R | TCCATGATATCCCACCGATT |
|  | DFR_4_F | AAACGCATCGTCTTCACCTC |
|  | DFR_4_R | ATGCAAATTCCGAACACACA |
| DFR1_SNP1 detection by pyrosequencing | FP_DFR1_SNP1 | TAAGCTAGAGCTGCTGCATGAA |
|  | BRP_DFR1_SNP1 | ATGCAAATTCCGAACACACA |
|  | SFP_DFR1_SNP1 | GAGCTGCTGCATGAA |
| DFR4_ID1 detection by pyrosequencing | SFP_DFR4_ID1 | GGCCTGCTGCAAGCAAAG |
|  | RP_DFR4_ID2 | AGGATCGAGTAGTGGGCTTCGTT |
|  | SRP_DFR4_ID3 | GACCCATCCACATGTTA |
