# Supplementary material for: Development of SNP markers for genes of the phenylpropanoid pathway and their association to kernel and malting traits in barley
Source: BMC Genet. 2013 Oct 2;14:97. doi: 10.1186/1471-2156-14-97 (PMC3852699; doi:10.1186/1471-2156-14-97)
Supplement: Additional file 11 — Used restriction enzymes for CAPS marker development and reaction conditions. [file 1471-2156-14-97-S11.docx]

Additional file 11: Used restriction enzymes for CAPS marker development and reaction conditions.

| Restriction enzyme | Manufactor | Buffer | Reaction temperature |
| --- | --- | --- | --- |
| *Acc*I | NEB | Puffer 3 | 37°C |
| *Taq*I | Fermentas | Taq-Puffer | 65 °C |
| *Hind*III | Fermentas | Puffer R | 37°C |
| *Mse*I | Fermentas | Puffer R | 65°C |
